# *N*-(3,5-Dichloro­phen­yl)maleamic acid

**DOI:** 10.1107/S1600536809051484

**Published:** 2009-12-04

**Authors:** B. Thimme Gowda, Miroslav Tokarčík, Jozef Kožíšek, K. Shakuntala, Hartmut Fuess

**Affiliations:** a Department of Chemistry, Mangalore University, Mangalagangotri 574 199, Mangalore, India; b Faculty of Chemical and Food Technology, Slovak Technical University, Radlinského 9, SK-812 37 Bratislava, Slovak Republic; c Institute of Materials Science, Darmstadt University of Technology, Petersenstrasse 23, D-64287, Darmstadt, Germany

## Abstract

In the title compound, C_10_H_7_Cl_2_NO_3_, the asymmetric unit contains four independent mol­ecules, which are linked to each other by N—H⋯O hydrogen bonds. The mol­ecular structure is stabilized by a short intra­molecular O—H⋯O hydrogen bond within each maleamic acid unit. In the crystal, the mol­ecules are linked into networks through N—H⋯O hydrogen bonds and inter­molecular C—Cl⋯O=C contacts [Cl⋯O = 3.0897 (12) and 3.0797 (13) Å].

## Related literature

For studies on the effect of ring- and side-chain substitutions on the crystal structures of amides, see: Gowda, Foro *et al.* (2009 ▶); Gowda, Tokarčík *et al.* (2009 ▶); Lo & Ng (2009 ▶); Prasad *et al.* (2002 ▶); Shakuntala *et al.* (2009 ▶). For short halogen–oxygen contacts, see: Fourmigué (2009 ▶). Kubicki (2004 ▶).
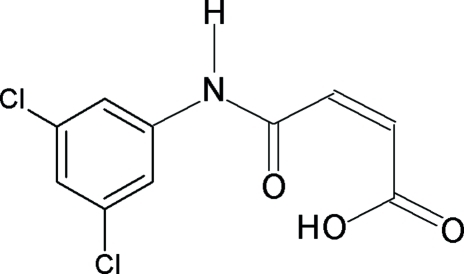


## Experimental

### 

#### Crystal data

C_10_H_7_Cl_2_NO_3_
*M* *_r_* = 260.07
Triclinic, 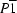

*a* = 8.13786 (12) Å
*b* = 16.5293 (3) Å
*c* = 17.4170 (3) Å
α = 103.4502 (17)°
β = 100.6466 (15)°
γ = 99.5964 (15)°
*V* = 2184.79 (7) Å^3^
*Z* = 8
Mo *K*α radiation
μ = 0.58 mm^−1^
*T* = 295 K
0.59 × 0.51 × 0.22 mm

#### Data collection

Oxford Diffraction Xcalibur Ruby Gemini diffractometer
Absorption correction: analytical (*CrysAlis PRO*, Oxford Diffraction, 2009 ▶) *T*_min_ = 0.728, *T*_max_ = 0.887
46919 measured reflections
8204 independent reflections
6694 reflections with *I* > 2σ(*I*)
*R*_int_ = 0.017

#### Refinement

*R*[*F* ^2^ > 2σ(*F* ^2^)] = 0.032
*wR*(*F* ^2^) = 0.088
*S* = 1.09
8204 reflections
581 parameters
H-atom parameters constrained
Δρ_max_ = 0.45 e Å^−3^
Δρ_min_ = −0.38 e Å^−3^

### 

Data collection: *CrysAlis PRO* (Oxford Diffraction, 2009 ▶); cell refinement: *CrysAlis PRO*; data reduction: *CrysAlis PRO*; program(s) used to solve structure: *SHELXS97* (Sheldrick, 2008 ▶); program(s) used to refine structure: *SHELXL97* (Sheldrick, 2008 ▶); molecular graphics: *ORTEP-3* (Farrugia, 1997 ▶) and *DIAMOND* (Brandenburg, 2002 ▶); software used to prepare material for publication: *SHELXL97*, *PLATON* (Spek, 2009 ▶) and *WinGX* (Farrugia, 1999 ▶).

## Supplementary Material

Crystal structure: contains datablocks I, global. DOI: 10.1107/S1600536809051484/dn2519sup1.cif

Structure factors: contains datablocks I. DOI: 10.1107/S1600536809051484/dn2519Isup2.hkl

Additional supplementary materials:  crystallographic information; 3D view; checkCIF report

## Figures and Tables

**Table 1:** Hydrogen-bond geometry (Å, °)

*D*—H⋯*A*	*D*—H	H⋯*A*	*D*⋯*A*	*D*—H⋯*A*
N11—H11⋯O33^i^	0.86	2.07	2.9254 (17)	172
N21—H21⋯O13	0.86	2.05	2.8748 (18)	161
N31—H31⋯O43	0.86	2.09	2.9244 (19)	165
N41—H41⋯O23	0.86	2.07	2.9186 (18)	168
O12—H12*A*⋯O11	0.82	1.65	2.4680 (18)	175
O22—H22*A*⋯O21	0.82	1.64	2.4613 (17)	177
O32—H32*A*⋯O31	0.82	1.66	2.4772 (17)	177
O42—H42*A*⋯O41	0.82	1.65	2.4684 (18)	172

Symmetry code: (i) 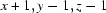.

**Fig. 1.**
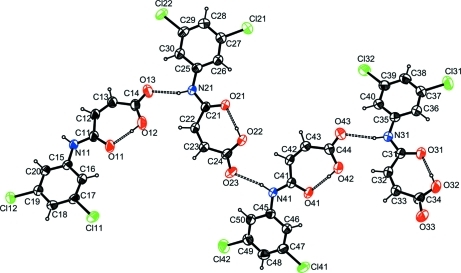
Molecular structure of (I) showing the atom labelling scheme. Displacement ellipsoids are drawn at the 30% probability level. H atoms are represented as small spheres of arbitrary radii. Hydrogen bonds are shown as dashed lines.

**Fig. 2.**
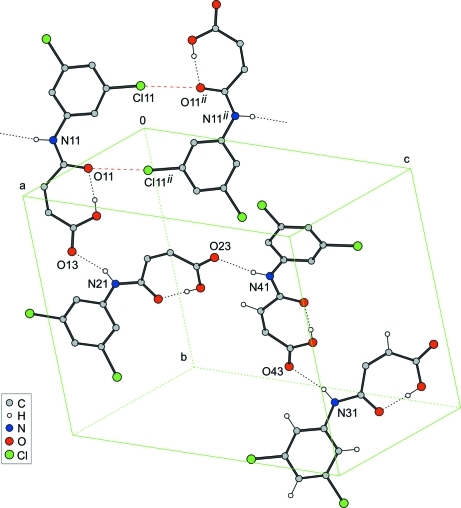
Part of crystal structure of (I) showing the chain of molecules linked by N–H···O hydrogen bonds (represented by dashed lines). The molecule with the amido atom N11 forms an inversion dimer stabilized via short Cl···O contacts involving the atoms Cl11 and O11.H atoms not involved in hydrogen bonding were omitted for clarity.[Symmetry code (ii): -x, -y, -z]

## supplementary crystallographic information

### Comment

In the present work, as a part of studying the effect of ring and side chain substitutions on the crystal structures of biologically significant amides (Gowda, Foro *et al.*,2009; Gowda, Tokarčík *et al.*, 2009; Shakuntala *et al.*, 2009; Prasad *et al.*, 2002), the crystal structure of *N*-(3,5-dichlorophenyl)maleamic acid (I) has been determined.

The asymmetric unit of (I) contains four independent molecules linked to each other through N-H···O intermolecular hydrogen bonds(Table 1, Fig. 1). The conformations of the N—H and C=O bonds in the amide segment of the structure are *anti* to each other and those of the amide O atom and the carbonyl O atom of the acid segment are also *anti* to each other. But the amide O atom is *anti* to the H atom attached to the adjacent C atom, while the carboxyl O atom is *syn* to the H atom attached to its adjacent C atom (Fig.1). In the structure of (I), relatively rare *anti* conformation of the C=O and O—H bonds of the acid group has been observed, similar to that obsrved in *N*-phenylmaleamic acid (Lo & Ng, 2009), *N*-(3,4-dimethylphenyl)maleamic acid, *N*-(2,4,6-trimethylphenyl)- maleamic acid (Gowda,Tokarčík *et al.*, 2009) and *N*-(2,5-dichlorophenyl)maleamic acid (Shakuntala *et al.*, 2009).

Each maleamic unit includes a short intramolecular hydrogen O—H···O bond (Table 1). Bond lengths C12–C13 =1.329 (2), C22–C23 =1.336 (2), C32–C33 =1.335 (2) and C42–C43 =1.329 (2)Å clearly indicate the double bond character.

The dihedral angles between the dichloro-substituted phenyl ring and the amido group –NHCO– are 4.5 (3), 8.4 (2), 10.4 (2) and 8.3 (3)° in the four independent molecules.

In the crystal structure, the intermolecular N–H···O hydrogen bonds link the molecules into infinite chain running parallel to the [-1 1 1] vector. The relatively short Cl···O contacts build up a two-dimensional network. Part of the crystal structure is shown in Fig. 2. The molecule containing the amido atom N11 forms an inversion dimer, which is is stabilized by two short Cl···O contacts with the length of 3.0897 (12)Å. Another short Cl···O contact between the atoms Cl12 and O41(iii) has the length of 3.0797 (13) Å. [Symmetry code (iii): *x*, *y*-1, *z*-1].

Our data for the C–Cl···O halogen bonds are in agreement with the observations of others (Kubicki, 2004; Fourmigué 2009).

### Experimental

The solution of maleic anhydride (0.025 mol) in toluene (25 ml) was treated dropwise with the solution of 3,5-dichloroaniline (0.025 mol) also in toluene (20 ml) with constant stirring. The resulting mixture was warmed with stirring for over 30 min and set aside for an additional 30 min at room temperature for completion of the reaction. The mixture was then treated with dilute hydrochloric acid to remove the unreacted 3,5-dichloroaniline. The resultant solid *N*-(3,5-dichlorophenyl)maleamic acid was filtered under suction and washed thoroughly with water to remove the unreacted maleic anhydride and maleic acid. It was recrystallized to constant melting point from ethanol. The purity of the compound was checked by elemental analysis and characterized by its infrared spectra. Colourless single crystals used in X-ray diffraction studies were grown in an ethanol solution by slow evaporation at room temperature.

### Refinement

All H atoms were visible in difference maps and further placed in calculated positions (C–H = 0.93 Å, N–H = 0.86 Å, N–H = 0.82 Å) and refined using the riding model. The *U*_iso_(H) values were set at 1.2*U*_eq_(C, N, O).

### Crystal data

**Table:**

C_10_H_7_Cl_2_NO_3_	*Z* = 8
*M**_r_* = 260.07	*F*(000) = 1056
Triclinic, *P*1	*D*_x_ = 1.581 Mg m^−^^3^
Hall symbol: -P 1	Mo *K*α radiation, λ = 0.71073 Å
*a* = 8.13786 (12) Å	Cell parameters from 29193 reflections
*b* = 16.5293 (3) Å	θ = 2.0–29.5°
*c* = 17.4170 (3) Å	µ = 0.58 mm^−^^1^
α = 103.4502 (17)°	*T* = 295 K
β = 100.6466 (15)°	Block, colourless
γ = 99.5964 (15)°	0.59 × 0.51 × 0.22 mm
*V* = 2184.79 (7) Å^3^	

### Data collection

**Table:**

Oxford Diffraction Xcalibur Ruby Gemini diffractometer	8204 independent reflections
graphite	6694 reflections with *I* > 2σ(*I*)
Detector resolution: 10.434 pixels mm^-1^	*R*_int_ = 0.017
ω scans	θ_max_ = 25.6°, θ_min_ = 2.0°
Absorption correction: analytical (*CrysAlis PRO*, Oxford Diffraction, 2009)	*h* = −9→9
*T*_min_ = 0.728, *T*_max_ = 0.887	*k* = −20→20
46919 measured reflections	*l* = −21→21

### Refinement

**Table:**

Refinement on *F*^2^	Primary atom site location: structure-invariant direct methods
Least-squares matrix: full	Secondary atom site location: difference Fourier map
*R*[*F*^2^ > 2σ(*F*^2^)] = 0.032	Hydrogen site location: inferred from neighbouring sites
*wR*(*F*^2^) = 0.088	H-atom parameters constrained
*S* = 1.09	*w* = 1/[σ^2^(*F*_o_^2^) + (0.0445*P*)^2^ + 0.4698*P*] where *P* = (*F*_o_^2^ + 2*F*_c_^2^)/3
8204 reflections	(Δ/σ)_max_ = 0.001
581 parameters	Δρ_max_ = 0.45 e Å^−^^3^
0 restraints	Δρ_min_ = −0.38 e Å^−^^3^

### Special details

**Table:**

Geometry. All esds (except the esd in the dihedral angle between two l.s. planes) are estimated using the full covariance matrix. The cell esds are taken into account individually in the estimation of esds in distances, angles and torsion angles; correlations between esds in cell parameters are only used when they are defined by crystal symmetry. An approximate (isotropic) treatment of cell esds is used for estimating esds involving l.s. planes.
Refinement. Refinement of *F*^2^ against ALL reflections. The weighted *R*-factor *wR* and goodness of fit *S* are based on *F*^2^, conventional *R*-factors *R* are based on *F*, with *F* set to zero for negative *F*^2^. The threshold expression of *F*^2^ > σ(*F*^2^) is used only for calculating *R*-factors(gt) *etc*. and is not relevant to the choice of reflections for refinement. *R*-factors based on *F*^2^ are statistically about twice as large as those based on *F*, and *R*- factors based on ALL data will be even larger.

### Fractional atomic coordinates and isotropic or equivalent isotropic displacement parameters (Å^2^)

**Table:**

	*x*	*y*	*z*	*U*_iso_*/*U*_eq_	
Cl11	−0.12991 (7)	−0.13131 (3)	−0.04861 (4)	0.07207 (17)	
Cl12	0.08943 (6)	−0.35296 (3)	−0.26076 (3)	0.05786 (14)	
O11	0.38762 (19)	0.06550 (8)	−0.04674 (10)	0.0728 (5)	
O12	0.51265 (18)	0.21919 (9)	0.01320 (10)	0.0688 (4)	
H12A	0.4669	0.1684	−0.0054	0.103*	
O13	0.72670 (19)	0.30909 (8)	0.00178 (9)	0.0716 (4)	
N11	0.42407 (17)	−0.04601 (8)	−0.14019 (8)	0.0403 (3)	
H11	0.4904	−0.0625	−0.1709	0.048*	
C11	0.4692 (2)	0.03579 (11)	−0.09563 (10)	0.0439 (4)	
C12	0.6215 (2)	0.08687 (11)	−0.10927 (11)	0.0473 (4)	
H12	0.6767	0.0576	−0.1453	0.057*	
C13	0.6907 (2)	0.16943 (11)	−0.07690 (11)	0.0499 (4)	
H13	0.7879	0.1886	−0.0941	0.060*	
C14	0.6420 (2)	0.23697 (11)	−0.01833 (11)	0.0465 (4)	
C15	0.2796 (2)	−0.10786 (10)	−0.14214 (9)	0.0369 (3)	
C16	0.1585 (2)	−0.08976 (11)	−0.09764 (11)	0.0448 (4)	
H16	0.1701	−0.0354	−0.0641	0.054*	
C17	0.0209 (2)	−0.15445 (11)	−0.10443 (11)	0.0449 (4)	
C18	−0.0017 (2)	−0.23577 (11)	−0.15278 (10)	0.0424 (4)	
H18	−0.0949	−0.2785	−0.1558	0.051*	
C19	0.1201 (2)	−0.25142 (10)	−0.19677 (10)	0.0391 (4)	
C20	0.2594 (2)	−0.18954 (10)	−0.19278 (10)	0.0382 (4)	
H20	0.3390	−0.2019	−0.2233	0.046*	
Cl21	0.84361 (9)	0.77742 (3)	0.13652 (4)	0.07855 (19)	
Cl22	0.89486 (6)	0.54211 (3)	−0.12381 (3)	0.05692 (13)	
O21	0.54916 (18)	0.52453 (8)	0.20423 (8)	0.0567 (3)	
O22	0.4351 (2)	0.49126 (8)	0.31739 (8)	0.0650 (4)	
H22A	0.4712	0.5037	0.2797	0.097*	
O23	0.36320 (18)	0.37768 (9)	0.35801 (8)	0.0615 (4)	
N21	0.63526 (17)	0.45503 (8)	0.09578 (8)	0.0396 (3)	
H21	0.6386	0.4052	0.0680	0.048*	
C21	0.5655 (2)	0.45722 (10)	0.16001 (10)	0.0391 (4)	
C22	0.5086 (2)	0.37266 (11)	0.17328 (10)	0.0420 (4)	
H22	0.5174	0.3261	0.1336	0.050*	
C23	0.4463 (2)	0.35426 (11)	0.23449 (10)	0.0428 (4)	
H23	0.4192	0.2963	0.2306	0.051*	
C24	0.4128 (2)	0.40956 (12)	0.30745 (10)	0.0446 (4)	
C25	0.7041 (2)	0.52430 (10)	0.06799 (10)	0.0381 (4)	
C26	0.7260 (2)	0.60907 (11)	0.11038 (10)	0.0436 (4)	
H26	0.6886	0.6236	0.1581	0.052*	
C27	0.8047 (2)	0.67118 (11)	0.07996 (11)	0.0488 (4)	
C28	0.8582 (2)	0.65292 (12)	0.00858 (11)	0.0491 (4)	
H28	0.9105	0.6959	−0.0108	0.059*	
C29	0.8309 (2)	0.56830 (12)	−0.03288 (10)	0.0428 (4)	
C30	0.7562 (2)	0.50368 (11)	−0.00454 (10)	0.0407 (4)	
H30	0.7407	0.4470	−0.0335	0.049*	
Cl31	0.55958 (9)	1.27986 (3)	0.76423 (5)	0.0959 (2)	
Cl32	0.82403 (7)	1.05115 (3)	0.58042 (4)	0.07005 (16)	
O31	0.12442 (17)	1.02362 (8)	0.74663 (8)	0.0571 (3)	
O32	−0.15047 (18)	0.99017 (9)	0.78660 (10)	0.0684 (4)	
H32A	−0.0581	1.0026	0.7750	0.103*	
O33	−0.36446 (17)	0.87993 (9)	0.75591 (9)	0.0645 (4)	
N31	0.25893 (18)	0.96191 (9)	0.65386 (9)	0.0436 (3)	
H31	0.2552	0.9152	0.6185	0.052*	
C31	0.1331 (2)	0.96077 (10)	0.69387 (10)	0.0411 (4)	
C32	0.0088 (2)	0.87797 (11)	0.67137 (10)	0.0433 (4)	
H32	0.0355	0.8333	0.6359	0.052*	
C33	−0.1363 (2)	0.85857 (11)	0.69501 (11)	0.0450 (4)	
H33	−0.1929	0.8016	0.6743	0.054*	
C34	−0.2235 (2)	0.91137 (12)	0.74824 (11)	0.0485 (4)	
C35	0.3974 (2)	1.03050 (10)	0.66258 (10)	0.0412 (4)	
C36	0.4083 (2)	1.11348 (11)	0.70717 (11)	0.0496 (4)	
H36	0.3236	1.1269	0.7340	0.060*	
C37	0.5476 (3)	1.17524 (11)	0.71059 (12)	0.0547 (5)	
C38	0.6779 (2)	1.15837 (12)	0.67291 (12)	0.0549 (5)	
H38	0.7719	1.2011	0.6770	0.066*	
C39	0.6626 (2)	1.07548 (11)	0.62901 (12)	0.0487 (4)	
C40	0.5251 (2)	1.01120 (11)	0.62243 (11)	0.0461 (4)	
H40	0.5174	0.9559	0.5918	0.055*	
Cl41	−0.02282 (7)	0.37735 (4)	0.72019 (3)	0.06395 (15)	
Cl42	0.24811 (9)	0.16293 (3)	0.52385 (4)	0.07410 (17)	
O41	0.1923 (2)	0.57709 (8)	0.57929 (9)	0.0737 (5)	
O42	0.2233 (2)	0.73192 (9)	0.59737 (10)	0.0750 (5)	
H42A	0.2170	0.6815	0.5957	0.112*	
O43	0.3060 (2)	0.81990 (8)	0.53013 (9)	0.0711 (4)	
N41	0.24994 (17)	0.46304 (8)	0.49887 (8)	0.0410 (3)	
H41	0.2850	0.4450	0.4557	0.049*	
C41	0.2470 (2)	0.54591 (10)	0.52001 (10)	0.0425 (4)	
C42	0.3126 (2)	0.59666 (11)	0.46873 (10)	0.0432 (4)	
H42	0.3481	0.5668	0.4248	0.052*	
C43	0.3275 (2)	0.67973 (11)	0.47709 (11)	0.0439 (4)	
H43	0.3736	0.6988	0.4377	0.053*	
C44	0.2847 (2)	0.74820 (11)	0.53737 (11)	0.0469 (4)	
C45	0.2013 (2)	0.40192 (10)	0.54029 (10)	0.0385 (4)	
C46	0.1217 (2)	0.41867 (11)	0.60458 (10)	0.0442 (4)	
H46	0.0955	0.4716	0.6220	0.053*	
C47	0.0823 (2)	0.35548 (12)	0.64202 (10)	0.0455 (4)	
C48	0.1202 (2)	0.27686 (12)	0.61936 (11)	0.0501 (4)	
H48	0.0949	0.2355	0.6462	0.060*	
C49	0.1980 (2)	0.26185 (11)	0.55458 (11)	0.0474 (4)	
C50	0.2381 (2)	0.32229 (11)	0.51464 (10)	0.0429 (4)	
H50	0.2892	0.3100	0.4710	0.051*	

### Atomic displacement parameters (Å^2^)

**Table:**

	*U*^11^	*U*^22^	*U*^33^	*U*^12^	*U*^13^	*U*^23^
Cl11	0.0741 (3)	0.0627 (3)	0.0933 (4)	0.0149 (3)	0.0646 (3)	0.0126 (3)
Cl12	0.0638 (3)	0.0411 (2)	0.0622 (3)	0.0011 (2)	0.0306 (2)	−0.0035 (2)
O11	0.0714 (9)	0.0484 (8)	0.0902 (10)	−0.0030 (7)	0.0571 (8)	−0.0163 (7)
O12	0.0658 (9)	0.0464 (8)	0.0823 (10)	0.0011 (6)	0.0405 (8)	−0.0155 (7)
O13	0.0817 (10)	0.0399 (8)	0.0843 (10)	−0.0005 (7)	0.0403 (8)	−0.0067 (7)
N11	0.0392 (7)	0.0373 (7)	0.0449 (8)	0.0084 (6)	0.0224 (6)	0.0021 (6)
C11	0.0439 (9)	0.0402 (9)	0.0457 (10)	0.0085 (7)	0.0197 (8)	0.0013 (7)
C12	0.0458 (9)	0.0426 (9)	0.0522 (10)	0.0093 (8)	0.0263 (8)	−0.0011 (8)
C13	0.0464 (10)	0.0436 (10)	0.0564 (11)	0.0031 (8)	0.0254 (9)	0.0015 (8)
C14	0.0483 (10)	0.0400 (10)	0.0467 (10)	0.0091 (8)	0.0138 (8)	0.0014 (8)
C15	0.0374 (8)	0.0384 (8)	0.0378 (8)	0.0094 (7)	0.0155 (7)	0.0097 (7)
C16	0.0499 (10)	0.0393 (9)	0.0482 (10)	0.0107 (7)	0.0257 (8)	0.0055 (7)
C17	0.0474 (9)	0.0476 (10)	0.0494 (10)	0.0155 (8)	0.0301 (8)	0.0137 (8)
C18	0.0420 (9)	0.0423 (9)	0.0464 (10)	0.0060 (7)	0.0195 (8)	0.0142 (8)
C19	0.0444 (9)	0.0367 (8)	0.0371 (9)	0.0101 (7)	0.0141 (7)	0.0073 (7)
C20	0.0389 (8)	0.0412 (9)	0.0383 (9)	0.0125 (7)	0.0176 (7)	0.0086 (7)
Cl21	0.1164 (5)	0.0404 (3)	0.0771 (4)	0.0009 (3)	0.0463 (3)	0.0053 (2)
Cl22	0.0648 (3)	0.0712 (3)	0.0456 (3)	0.0192 (2)	0.0299 (2)	0.0205 (2)
O21	0.0796 (9)	0.0398 (7)	0.0569 (8)	0.0105 (6)	0.0441 (7)	0.0048 (6)
O22	0.0947 (11)	0.0524 (8)	0.0509 (8)	0.0086 (7)	0.0460 (8)	0.0027 (6)
O23	0.0785 (9)	0.0694 (9)	0.0479 (7)	0.0176 (7)	0.0363 (7)	0.0195 (7)
N21	0.0481 (8)	0.0355 (7)	0.0384 (7)	0.0110 (6)	0.0213 (6)	0.0062 (6)
C21	0.0393 (8)	0.0412 (9)	0.0369 (8)	0.0082 (7)	0.0161 (7)	0.0053 (7)
C22	0.0486 (9)	0.0392 (9)	0.0395 (9)	0.0103 (7)	0.0207 (8)	0.0046 (7)
C23	0.0453 (9)	0.0417 (9)	0.0434 (9)	0.0087 (7)	0.0185 (8)	0.0096 (7)
C24	0.0418 (9)	0.0541 (11)	0.0377 (9)	0.0076 (8)	0.0163 (7)	0.0087 (8)
C25	0.0348 (8)	0.0431 (9)	0.0390 (9)	0.0095 (7)	0.0124 (7)	0.0124 (7)
C26	0.0469 (9)	0.0433 (9)	0.0414 (9)	0.0086 (7)	0.0183 (8)	0.0079 (7)
C27	0.0556 (11)	0.0399 (9)	0.0498 (10)	0.0067 (8)	0.0178 (9)	0.0083 (8)
C28	0.0523 (10)	0.0485 (10)	0.0506 (11)	0.0068 (8)	0.0193 (9)	0.0186 (8)
C29	0.0386 (9)	0.0576 (11)	0.0378 (9)	0.0142 (8)	0.0152 (7)	0.0160 (8)
C30	0.0422 (9)	0.0440 (9)	0.0377 (9)	0.0131 (7)	0.0137 (7)	0.0087 (7)
Cl31	0.1081 (5)	0.0399 (3)	0.1330 (6)	−0.0019 (3)	0.0686 (4)	−0.0086 (3)
Cl32	0.0627 (3)	0.0547 (3)	0.1095 (5)	0.0189 (2)	0.0499 (3)	0.0271 (3)
O31	0.0623 (8)	0.0436 (7)	0.0635 (8)	0.0072 (6)	0.0343 (7)	−0.0014 (6)
O32	0.0652 (9)	0.0518 (8)	0.0915 (11)	0.0138 (7)	0.0504 (8)	0.0001 (7)
O33	0.0522 (8)	0.0674 (9)	0.0782 (10)	0.0122 (7)	0.0375 (7)	0.0113 (7)
N31	0.0513 (8)	0.0350 (7)	0.0469 (8)	0.0084 (6)	0.0234 (7)	0.0070 (6)
C31	0.0454 (9)	0.0399 (9)	0.0425 (9)	0.0141 (7)	0.0181 (8)	0.0101 (7)
C32	0.0480 (9)	0.0381 (9)	0.0457 (9)	0.0127 (7)	0.0201 (8)	0.0058 (7)
C33	0.0466 (9)	0.0407 (9)	0.0484 (10)	0.0093 (7)	0.0191 (8)	0.0076 (8)
C34	0.0488 (10)	0.0524 (11)	0.0527 (11)	0.0176 (8)	0.0249 (9)	0.0153 (9)
C35	0.0481 (9)	0.0390 (9)	0.0409 (9)	0.0102 (7)	0.0163 (7)	0.0143 (7)
C36	0.0581 (11)	0.0413 (9)	0.0537 (11)	0.0109 (8)	0.0260 (9)	0.0106 (8)
C37	0.0675 (12)	0.0364 (9)	0.0604 (12)	0.0077 (8)	0.0254 (10)	0.0075 (8)
C38	0.0542 (11)	0.0417 (10)	0.0703 (13)	0.0037 (8)	0.0220 (10)	0.0172 (9)
C39	0.0502 (10)	0.0453 (10)	0.0608 (11)	0.0153 (8)	0.0240 (9)	0.0217 (9)
C40	0.0529 (10)	0.0376 (9)	0.0540 (11)	0.0131 (8)	0.0221 (8)	0.0143 (8)
Cl41	0.0705 (3)	0.0751 (3)	0.0505 (3)	0.0069 (3)	0.0327 (2)	0.0171 (2)
Cl42	0.1134 (5)	0.0526 (3)	0.0719 (4)	0.0381 (3)	0.0316 (3)	0.0251 (3)
O41	0.1289 (13)	0.0460 (8)	0.0721 (9)	0.0321 (8)	0.0727 (10)	0.0180 (7)
O42	0.1258 (13)	0.0440 (8)	0.0763 (10)	0.0302 (9)	0.0658 (10)	0.0158 (7)
O43	0.1044 (12)	0.0400 (8)	0.0795 (10)	0.0188 (7)	0.0456 (9)	0.0158 (7)
N41	0.0503 (8)	0.0369 (7)	0.0386 (7)	0.0099 (6)	0.0229 (6)	0.0058 (6)
C41	0.0498 (10)	0.0388 (9)	0.0419 (9)	0.0124 (7)	0.0206 (8)	0.0070 (7)
C42	0.0509 (10)	0.0430 (9)	0.0402 (9)	0.0142 (8)	0.0226 (8)	0.0077 (7)
C43	0.0477 (9)	0.0433 (9)	0.0453 (10)	0.0106 (7)	0.0200 (8)	0.0135 (8)
C44	0.0528 (10)	0.0375 (10)	0.0507 (10)	0.0097 (8)	0.0177 (8)	0.0083 (8)
C45	0.0377 (8)	0.0378 (8)	0.0381 (9)	0.0043 (7)	0.0111 (7)	0.0077 (7)
C46	0.0455 (9)	0.0420 (9)	0.0443 (9)	0.0063 (7)	0.0175 (8)	0.0070 (8)
C47	0.0408 (9)	0.0547 (11)	0.0373 (9)	0.0012 (8)	0.0125 (7)	0.0094 (8)
C48	0.0530 (10)	0.0519 (11)	0.0455 (10)	0.0040 (8)	0.0106 (8)	0.0194 (8)
C49	0.0526 (10)	0.0426 (9)	0.0461 (10)	0.0119 (8)	0.0090 (8)	0.0110 (8)
C50	0.0453 (9)	0.0436 (9)	0.0392 (9)	0.0098 (7)	0.0135 (7)	0.0072 (7)

### Geometric parameters (Å, °)

**Table:**

Cl11—C17	1.7383 (15)	Cl31—C37	1.7410 (18)
Cl12—C19	1.7347 (16)	Cl32—C39	1.7412 (18)
O11—C11	1.2370 (19)	O31—C31	1.2383 (19)
O12—C14	1.297 (2)	O32—C34	1.299 (2)
O12—H12A	0.8200	O32—H32A	0.8200
O13—C14	1.209 (2)	O33—C34	1.222 (2)
N11—C11	1.344 (2)	N31—C31	1.341 (2)
N11—C15	1.413 (2)	N31—C35	1.417 (2)
N11—H11	0.8600	N31—H31	0.8600
C11—C12	1.469 (2)	C31—C32	1.481 (2)
C12—C13	1.329 (2)	C32—C33	1.335 (2)
C12—H12	0.9300	C32—H32	0.9300
C13—C14	1.487 (2)	C33—C34	1.483 (2)
C13—H13	0.9300	C33—H33	0.9300
C15—C16	1.390 (2)	C35—C36	1.390 (2)
C15—C20	1.395 (2)	C35—C40	1.395 (2)
C16—C17	1.380 (2)	C36—C37	1.375 (3)
C16—H16	0.9300	C36—H36	0.9300
C17—C18	1.372 (2)	C37—C38	1.380 (3)
C18—C19	1.382 (2)	C38—C39	1.378 (3)
C18—H18	0.9300	C38—H38	0.9300
C19—C20	1.374 (2)	C39—C40	1.376 (2)
C20—H20	0.9300	C40—H40	0.9300
Cl21—C27	1.7462 (18)	Cl41—C47	1.7382 (17)
Cl22—C29	1.7400 (16)	Cl42—C49	1.7383 (18)
O21—C21	1.2399 (19)	O41—C41	1.236 (2)
O22—C24	1.298 (2)	O42—C44	1.301 (2)
O22—H22A	0.8200	O42—H42A	0.8200
O23—C24	1.222 (2)	O43—C44	1.208 (2)
N21—C21	1.341 (2)	N41—C41	1.339 (2)
N21—C25	1.415 (2)	N41—C45	1.417 (2)
N21—H21	0.8600	N41—H41	0.8600
C21—C22	1.482 (2)	C41—C42	1.470 (2)
C22—C23	1.336 (2)	C42—C43	1.329 (2)
C22—H22	0.9300	C42—H42	0.9300
C23—C24	1.482 (2)	C43—C44	1.490 (2)
C23—H23	0.9300	C43—H43	0.9300
C25—C26	1.388 (2)	C45—C50	1.389 (2)
C25—C30	1.392 (2)	C45—C46	1.391 (2)
C26—C27	1.380 (2)	C46—C47	1.379 (2)
C26—H26	0.9300	C46—H46	0.9300
C27—C28	1.377 (2)	C47—C48	1.371 (3)
C28—C29	1.376 (2)	C48—C49	1.386 (3)
C28—H28	0.9300	C48—H48	0.9300
C29—C30	1.376 (2)	C49—C50	1.374 (2)
C30—H30	0.9300	C50—H50	0.9300
			
C14—O12—H12A	109.5	C34—O32—H32A	109.5
C11—N11—C15	127.15 (13)	C31—N31—C35	128.06 (14)
C11—N11—H11	116.4	C31—N31—H31	116.0
C15—N11—H11	116.4	C35—N31—H31	116.0
O11—C11—N11	122.01 (15)	O31—C31—N31	122.38 (16)
O11—C11—C12	122.74 (15)	O31—C31—C32	122.95 (15)
N11—C11—C12	115.25 (13)	N31—C31—C32	114.65 (14)
C13—C12—C11	128.48 (15)	C33—C32—C31	128.94 (15)
C13—C12—H12	115.8	C33—C32—H32	115.5
C11—C12—H12	115.8	C31—C32—H32	115.5
C12—C13—C14	132.18 (16)	C32—C33—C34	131.68 (17)
C12—C13—H13	113.9	C32—C33—H33	114.2
C14—C13—H13	113.9	C34—C33—H33	114.2
O13—C14—O12	119.44 (16)	O33—C34—O32	120.35 (16)
O13—C14—C13	119.61 (16)	O33—C34—C33	119.38 (17)
O12—C14—C13	120.92 (15)	O32—C34—C33	120.27 (15)
C16—C15—C20	120.07 (15)	C36—C35—C40	120.31 (16)
C16—C15—N11	122.84 (14)	C36—C35—N31	123.30 (15)
C20—C15—N11	117.07 (13)	C40—C35—N31	116.38 (15)
C17—C16—C15	118.37 (15)	C37—C36—C35	118.29 (16)
C17—C16—H16	120.8	C37—C36—H36	120.9
C15—C16—H16	120.8	C35—C36—H36	120.9
C18—C17—C16	123.10 (15)	C36—C37—C38	123.09 (17)
C18—C17—Cl11	118.80 (13)	C36—C37—Cl31	118.59 (14)
C16—C17—Cl11	118.10 (13)	C38—C37—Cl31	118.31 (14)
C17—C18—C19	117.05 (15)	C39—C38—C37	117.04 (17)
C17—C18—H18	121.5	C39—C38—H38	121.5
C19—C18—H18	121.5	C37—C38—H38	121.5
C20—C19—C18	122.50 (15)	C40—C39—C38	122.49 (16)
C20—C19—Cl12	119.90 (12)	C40—C39—Cl32	118.83 (14)
C18—C19—Cl12	117.58 (12)	C38—C39—Cl32	118.68 (14)
C19—C20—C15	118.89 (14)	C39—C40—C35	118.75 (16)
C19—C20—H20	120.6	C39—C40—H40	120.6
C15—C20—H20	120.6	C35—C40—H40	120.6
C24—O22—H22A	109.5	C44—O42—H42A	109.5
C21—N21—C25	128.20 (14)	C41—N41—C45	126.76 (13)
C21—N21—H21	115.9	C41—N41—H41	116.6
C25—N21—H21	115.9	C45—N41—H41	116.6
O21—C21—N21	122.61 (15)	O41—C41—N41	121.40 (16)
O21—C21—C22	122.90 (14)	O41—C41—C42	122.65 (15)
N21—C21—C22	114.48 (13)	N41—C41—C42	115.95 (13)
C23—C22—C21	128.81 (15)	C43—C42—C41	128.27 (15)
C23—C22—H22	115.6	C43—C42—H42	115.9
C21—C22—H22	115.6	C41—C42—H42	115.9
C22—C23—C24	131.60 (16)	C42—C43—C44	132.34 (16)
C22—C23—H23	114.2	C42—C43—H43	113.8
C24—C23—H23	114.2	C44—C43—H43	113.8
O23—C24—O22	120.20 (16)	O43—C44—O42	119.42 (16)
O23—C24—C23	119.55 (17)	O43—C44—C43	119.52 (17)
O22—C24—C23	120.24 (15)	O42—C44—C43	121.06 (16)
C26—C25—C30	120.11 (15)	C50—C45—C46	119.75 (15)
C26—C25—N21	123.43 (14)	C50—C45—N41	117.07 (14)
C30—C25—N21	116.42 (14)	C46—C45—N41	123.17 (15)
C27—C26—C25	118.29 (15)	C47—C46—C45	118.83 (16)
C27—C26—H26	120.9	C47—C46—H46	120.6
C25—C26—H26	120.9	C45—C46—H46	120.6
C28—C27—C26	123.00 (16)	C48—C47—C46	122.88 (16)
C28—C27—Cl21	118.79 (14)	C48—C47—Cl41	119.41 (14)
C26—C27—Cl21	118.18 (13)	C46—C47—Cl41	117.70 (14)
C29—C28—C27	117.17 (16)	C47—C48—C49	116.89 (16)
C29—C28—H28	121.4	C47—C48—H48	121.6
C27—C28—H28	121.4	C49—C48—H48	121.6
C30—C29—C28	122.29 (15)	C50—C49—C48	122.50 (16)
C30—C29—Cl22	118.87 (13)	C50—C49—Cl42	119.14 (14)
C28—C29—Cl22	118.84 (13)	C48—C49—Cl42	118.36 (14)
C29—C30—C25	119.10 (15)	C49—C50—C45	119.13 (15)
C29—C30—H30	120.4	C49—C50—H50	120.4
C25—C30—H30	120.4	C45—C50—H50	120.4

### Hydrogen-bond geometry (Å, °)

**Table:**

*D*—H···*A*	*D*—H	H···*A*	*D*···*A*	*D*—H···*A*
N11—H11···O33^i^	0.86	2.07	2.9254 (17)	172
N21—H21···O13	0.86	2.05	2.8748 (18)	161
N31—H31···O43	0.86	2.09	2.9244 (19)	165
N41—H41···O23	0.86	2.07	2.9186 (18)	168
O12—H12A···O11	0.82	1.65	2.4680 (18)	175
O22—H22A···O21	0.82	1.64	2.4613 (17)	177
O32—H32A···O31	0.82	1.66	2.4772 (17)	177
O42—H42A···O41	0.82	1.65	2.4684 (18)	172

Symmetry codes: (i) *x*+1, *y*−1, *z*−1.
